# Post-marketing safety surveillance and signal characterization of the novel dissociative steroid Vamorolone in Duchenne muscular dystrophy: a comparative disproportionality analysis based on FAERS data

**DOI:** 10.3389/fphar.2026.1838974

**Published:** 2026-06-26

**Authors:** Yining Zeng, Hao Wu, Xueting Wang, Xiyue Tan, Ruixia Wang, Yuxia Liu, Junguo Duan

**Affiliations:** 1 Eye School of Chengdu University of TCM, Chengdu, Sichuan, China; 2 Ineye Hospital of Chengdu University of TCM, Chengdu, Sichuan, China; 3 Eye Health with Traditional Chinese Medicine Key Laboratory of Sichuan Province, Chengdu, Sichuan, China

**Keywords:** adverse drug events, duchenne muscular dystrophy, FAERS, pharmacovigilance, vamorolone

## Abstract

**Objective:**

To comprehensively evaluate the real-world post-marketing safety profile of the novel dissociative steroid Vamorolone and perform a comparative analysis against the traditional glucocorticoid deflazacort.

**Methods:**

Vamorolone-associated adverse event reports were extracted from the FAERS database (2023Q4–2025Q4) without indication restrictions to maximize detection sensitivity. Disproportionality analysis utilized ROR, PRR, BCPNN, and MGPS, reinforced by a strict Bonferroni correction to assess statistical robustness against multiple testing. Signal novelty was systematically assessed against the official FDA prescribing information. Temporal onset was evaluated via the Weibull distribution. To rigorously compare safety profiles across skeletal, growth, endocrine, and psychiatric domains, an exploratory head-to-head analysis was conducted using 2 × 2 contingency tables to calculate crude odds ratios against deflazacort.

**Results:**

We identified 1,171 cases (97.0% explicitly confirmed for muscular dystrophy) comprising 2,860 reported adverse events. Multi-algorithm consensus screening yielded a final refined cohort of 60 potential adverse event signals, predominantly concentrated within psychiatric disorders, investigations, and infections. Label cross-referencing revealed known risks (e.g., stubbornness, EBGM = 163.78 and acute adrenocortical insufficiency, EBGM = 71.14) and potentially novel signals (e.g., enuresis, EBGM = 42.40 and troponin I increased, EBGM = 39.54) that maintained statistical robustness under the stringent Bonferroni threshold. AEs exhibited an “early failure” onset (β = 0.74), particularly rapid for psychiatric events (median 36.5 days, β = 0.57). Comparatively, Vamorolone showed significantly lower reporting odds than deflazacort for growth retardation (Crude OR = 0.15, P = 0.003), osteoporosis (Crude OR = 0.27, P = 0.02), and femur fracture (Crude OR = 0.38, P = 0.008), while psychiatric signals remained comparable (P > 0.05).

**Conclusion:**

Exploratory analyses generate the hypothesis that Vamorolone may be associated with lower reporting odds for skeletal and growth-related adverse events compared to deflazacort. Nevertheless, known risks and unexpected hypothesis-generating signals—such as notable HPA axis suppression and early-onset psychiatric disturbances—remain critical safety concerns. Clinicians should interpret these differential reporting patterns with caution and rigorously monitor neurobehavioral and adrenal health during the first three months, with further external validation warranted.

## 1 Introduction

Duchenne muscular dystrophy (DMD), a rare X-linked recessive neuromuscular disease, is caused by mutations in the gene encoding dystrophin (Romitti et al., 2015; Salari et al., 2022). This deficiency leads to the absence of the protein at the sarcolemma, subsequently triggering progressive muscle cell damage, chronic inflammation, fibrosis, and ultimately, respiratory and circulatory failure (Kohler et al., 2009; Flanigan, 2014; Deconinck and Dan, 2007). While traditional glucocorticoids (GCs)—such as prednisone and deflazacort—remain the cornerstone of DMD management by prolonging ambulation and preserving cardiopulmonary function (Crisafulli et al., 2020), their long-term utility is severely limited by multi-system toxicities (Griggs et al., 2016; McDonald et al., 2018). These adverse effects, including marked growth inhibition, osteoporosis, Cushingoid features, and behavioral disorders, frequently necessitate dosage reductions or treatment discontinuation (Bello et al., 2015; Guglieri et al., 2022; Vandewalle et al., 2018).

To address this critical unmet need, Vamorolone (Agamree) was developed as a first-in-class “dissociative steroid.” Of note, activation of the proinflammatory nuclear factor-κB (NF-κB) cascade is detectable in the skeletal muscle of DMD patients as early as birth, and this aberrant signaling is thought to trigger persistent muscle inflammation that drives the initiation and advancement of the disease (Guiraud et al., 2015; Rosenberg et al., 2015). Unlike traditional 11*β*-hydroxysteroids, Vamorolone features a unique Δ9,11 structural modification that selectively preserves the anti-inflammatory “transrepression” function (e.g., inhibiting NF-κB) while substantially attenuating the “transactivation” pathways responsible for GC-associated toxicities (Vandewalle et al., 2018; Heier et al., 2019; Reeves et al., 2013).

Based on the robust findings of the pivotal Phase IIb VISION-DMD clinical trial, Vamorolone was approved by the U.S. FDA in October 2023 for the treatment of DMD in patients aged 2 years and older (Dang et al., 2024). Clinical data indicates that while preserving motor function, its impact on linear growth and bone metabolism is significantly more favorable than that of prednisone (Dang et al., 2024). However, despite encouraging clinical trial results, inherent limitations such as restricted sample sizes, limited observation periods, and strict inclusion criteria often preclude a comprehensive understanding of a drug’s full safety profile in widespread real-world applications (Potter et al., 2025). This is particularly true for delayed-onset or low-frequency adverse events within rare disease populations. The FDA Adverse Event Reporting System (FAERS), which aggregates large-scale spontaneous reporting data globally, provides a crucial avenue for evaluating the early safety signals of newly marketed drugs (Potter et al., 2025). As Vamorolone is progressively introduced to the global market, systematically evaluating its performance in real-world clinical settings and conducting an in-depth comparative safety analysis against traditional gold-standard therapies holds profound academic and practical value.

Therefore, this study utilizes large-scale FAERS data to conduct a disproportionality analysis, systematically delineating the post-marketing adverse event spectrum of Vamorolone and comparing it against traditional gold-standard therapies. Ultimately, this analysis aims to comprehensively evaluate the real-world safety profile of Vamorolone and verify whether its anticipated safety advantages are reflected in post-marketing populations, thereby providing robust evidence to guide precision prescribing and risk management in DMD.

## 2 Methods

### 2.1 Date source

This study relies on data sourced from FAERS, a publicly available pharmacovigilance database. As a vital tool for monitoring medication safety, FAERS is a professionally maintained repository that compiles adverse events (AEs) reports worldwide from a variety of contributors, including patients, medical professionals, and drug manufacturers. The database is updated every quarter and organized into seven fundamental files: patient demographics, drug information, adverse event terms, clinical outcomes, reporting sources, therapy dates, and indications for use. Because Vamorolone (AGAMREE) received U.S. approval on 26 October 2023, the analysis was restricted to reports submitted from 2023Q4 - 2025Q4. To identify relevant cases, we applied the generic name “VAMOROLONE” along with its trade name “AGAMREE” as the core search terms, finalizing data extraction on 1 March 2026. Because the acquired files are entirely de-identified and devoid of personal health information, ethical board approval was not required.

### 2.2 Data extraction and analysis

**FIGURE 1:**
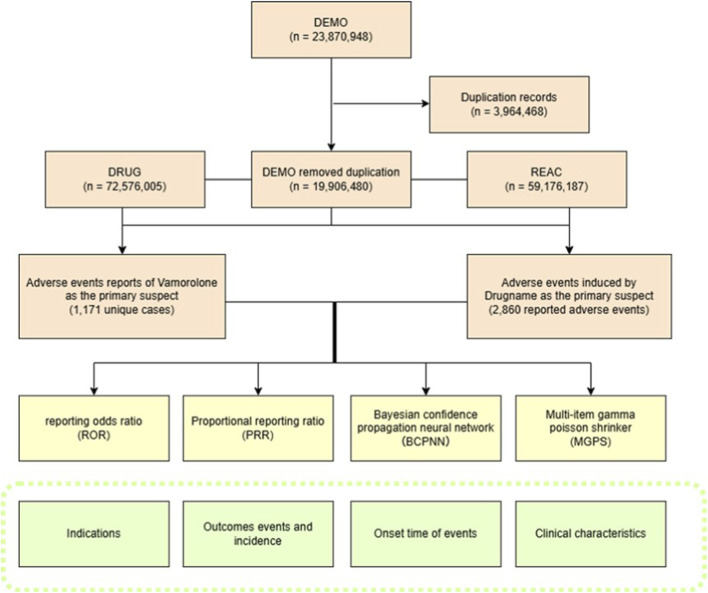
Flowchart of the data extraction and signal detection process from the FAERS database.

To ensure the integrity of our dataset and accurate calculation of background reporting rates, a rigorous data cleaning protocol was implemented as the initial preprocessing step, strictly in accordance with FDA recommendations. First, all invalid or flagged records—identified systematically via the quarterly deletion tables (DELETE files) published by the FDA—were completely removed from the baseline cohort. Subsequently, a global database-level deduplication was performed: when multiple reports existed, only the version bearing the latest FDA receipt date (FDA_DT) was preserved. In scenarios where both the FDA_DT and CASE_ID were identical, the record with the largest PRIMARY_ID was chosen for downstream analysis. Furthermore, the analysis was restricted to reports containing a minimum of three Preferred Terms (PTs). Given that raw FAERS data files contain chronological variations in MedDRA coding, all extracted adverse event terms were retrospectively mapped and standardized to a single, unified version (MedDRA version 28.1) to ensure hierarchical consistency and accurate PT-to-SOC alignment across the entire study period. We categorized these events by their respective PTs and System Organ Classes (SOCs) to conduct a thorough evaluation of Vamorolone-related safety signals. This comprehensive evaluation covered various clinical aspects, including patient demographics (age and sex), the timeline of AE occurrence, event types, severity, and other pertinent clinical characteristics. Upon completing the preprocessing phase, the refined dataset consisted of 19,906,480 demographic (DEMO) records, 72,576,005 medication (DRUG) records, and 59,176,187 reaction (REAC) records. Subsequently, we queried this fully deduplicated database for our specific target. A total of 1,171 unique FAERS cases (CASE_ID-level reports in which Vamorolone was coded as the primary suspect drug) were successfully identified and extracted. To ensure maximum sensitivity in capturing post-marketing safety signals for this newly approved orphan drug, all spontaneous reports involving Vamorolone were retained without restricting by indication. A subsequent review of the indication files confirmed that 97.0% (1,136 of 1,171 cases) of the reports were specifically labeled for Duchenne muscular dystrophy or general muscular dystrophy. The remaining 3.0% (35 cases) representing missing or off-label uses were retained to prevent signal loss. These cases comprised 2,860 PT-level reaction records in total, because a single FAERS case may contain multiple reported adverse events (Figure 1).

### 2.3 Statistical analysis

#### 2.3.1 Disproportionality analysis and signal detection

To evaluate the correlation between Vamorolone and adverse events, descriptive statistics were combined with disproportionality analysis, the established gold standard for data mining in spontaneous reporting systems. Our methodology utilized four distinct signal detection algorithms: the Reporting Odds Ratio (ROR) (van Puijenbroek et al., 2002), Proportional Reporting Ratio (PRR) (Evans et al., 2001), Bayesian Confidence Propagation Neural Network (BCPNN) (Bate et al., 1998), and the Multi-Item Gamma Poisson Shrinker (MGPS) (Dumouchel, 1999). To maximize the detection sensitivity, the entire FAERS database during the study timeframe served as the background comparator. Considering that this novel drug has been on the market for only 2 years, we established a global minimum inclusion threshold of at least 3 reports (N ≥ 3) (Evans et al., 2001; Harpaz et al., 2012). To control the false discovery rate and mitigate the inherent statistical noise in large spontaneous reporting databases, a conservative consensus framework was implemented. A PT was recognized as a positive safety signal only if it concurrently satisfied the pre-specified criteria of all four algorithms, a rigorous multi-method approach widely adopted to enhance signal specificity in pharmacovigilance studies (Harpaz et al., 2012; Szarfman et al., 2002): the lower 95% confidence limit of the ROR > 1; PRR ≥ 2 with χ^2^ ≥ 4; the lower limit of the 95% confidence interval of the Information Component (IC025) > 0; and the lower limit of the one-sided 95% confidence interval (the 5th percentile) of the Empirical Bayes Geometric Mean (EBGM05) > 2. ROR was used as the primary descriptive measure in the main tables, whereas PRR, BCPNN, and MGPS were used to assess cross-method consistency. By requiring simultaneous positivity across all four algorithms, our consensus approach integrates the sensitivity of frequentist models with the specificity of Bayesian models. Detailed mathematical equations and the complete 2 × 2 contingency matrix are provided in Supplementary Table S1. Moreover, to classify the detected adverse event signals as “expected” (labeled) or “unexpected” (unlabeled/novel), we systematically cross-referenced all positive signals against the official U.S. FDA approved prescribing information for AGAMREE (Vamorolone), revised in June 2024 (Catalyst Pharmaceuticals, 2023).

#### 2.3.2 Time-to-onset and weibull distribution analysis

Time-to-onset (TTO) was calculated as the time interval (in days) between the start date of Vamorolone therapy (START_DT) and the date of adverse event onset (EVENT_DT). Cases with missing dates or implausible chronological records (e.g., EVENT_DT preceding START_DT) were strictly excluded from this specific analysis. To characterize the temporal hazard profile, the Weibull shape (β) and scale (α) parameters, alongside their 95% confidence intervals, were estimated utilizing the maximum likelihood estimation method.

#### 2.3.3 Comparative safety analysis

In addition to the overall safety profiling, an exploratory comparative safety analysis was executed against deflazacort, a conventional therapy for Duchenne muscular dystrophy. This comparison systematically evaluated safety signals across four critical domains: growth and development, bone health, endocrine function, and psychiatric manifestations. To directly compare the relative reporting frequencies, the comparative cohort was restricted to cases where Vamorolone or deflazacort was identified as the Primary Suspect. We constructed head-to-head 2 × 2 contingency tables and estimated the comparative relative risk using the crude odds ratio (Crude OR) with 95% confidence intervals (CIs). Statistical significance (*P*-value) was determined using the Pearson chi-square test, or Fisher’s exact test when expected cell counts were less than 5. It is important to note that this comparative analysis relies on crude spontaneous reporting data without adjustment for potential confounders such as age, disease severity, or prior corticosteroid exposure. Therefore, this component of the study is strictly exploratory and intended solely for hypothesis generation regarding the relative safety profiles.

#### 2.3.4 Multiple-testing robustness assessment

To rigorously evaluate the internal robustness of our findings and address the inherent risk of false-positive signals generated by large-scale multiple testing in pharmacovigilance databases, a sensitivity analysis was conducted. We applied the ultra-conservative Bonferroni correction to our primary disproportionality results. The threshold for statistical significance was adjusted to a strictly penalized *P*-value using the formula α = 0.05/N, where N represents the total number of unique PTs associated with Vamorolone evaluated in the dataset. Signals that maintained statistical significance under this stringent criterion were considered highly robust.

The core data preprocessing, cleaning, and pipeline management were executed using Python (version 3.10) in conjunction with the Pandas (version 2.0) and NumPy (version 1.24) libraries. All subsequent statistical computations were performed utilizing MySQL 8.0 and Microsoft Excel 2019, while data visualization was rendered through R software (version 4.3.1). The study followed the READUS-PV guidance for disproportionality analyses, with the completed checklist provided in the Supplementary Material.

## 3 Results

### 3.1 Basic information on adverse events of vamorolone

**FIGURE 2:**
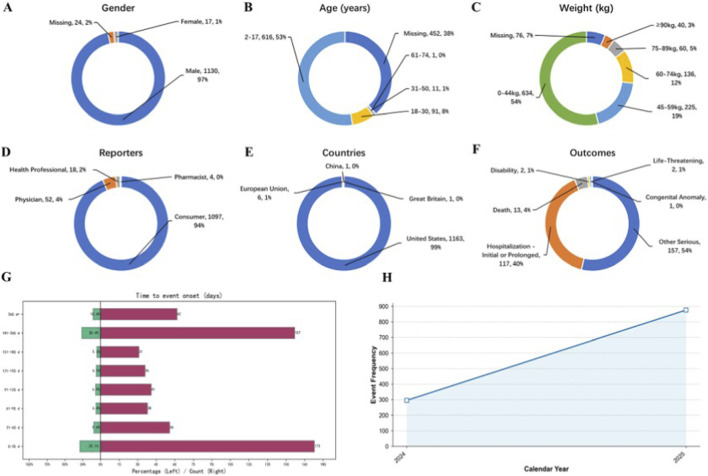
Demographic and clinical characteristics of Vamorolone-associated adverse event reports. **(A)** Gender Distribution; **(B)** Age Distribution; **(C)** Weight Distribution; **(D)** Reporter Distribution; **(E)** Reporting Country Distribution; **(F)** Outcome Distribution; **(G)** Time to Onset Distribution; **(H)** Reporting Time Series.

**FIGURE 3:**
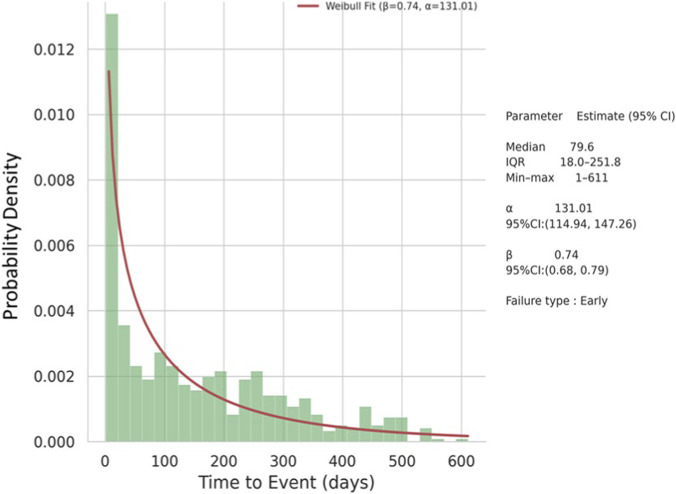
Time-to-onset profile and Weibull distribution shape analysis for overall Vamorolone-associated adverse event signals. Note: Time-to-Event: Represents the time interval (in days) from the initiation of therapy to the first occurrence of the adverse event. Probability Density: Reflects the relative temporal likelihood or concentration of event onset, rather than the true clinical incidence rate.

Figure 2 summarizes the demographic and clinical profiles of Vamorolone-associated adverse event reports. Consistent with the typical DMD patient population, the cohort was overwhelmingly male (97.0%, n = 1,130) and predominantly pediatric, with the majority aged 2–17 years (53.0%) and weighing 0–44 kg (54.0%). The vast majority of these reports originated from the United States (99.0%) and were primarily submitted by consumers (94.0%). Regarding clinical outcomes, “Other Serious” events (54.0%) and “Hospitalization” (40.0%) constituted the bulk of the severity records. TTO analysis revealed that AEs occurred most frequently within the first month of treatment (0–30 days, 29.1%), followed by a second peak between 181 and 360 days (26.4%). Additionally, longitudinal tracking demonstrated a continuous upward trend in the frequency of AE reports from 2024 to 2025. The TTO characteristics of Vamorolone-associated AEs are detailed in Figure 3. After excluding reports with missing or implausible dates, exactly 594 primary suspect cases (50.7%) possessed valid temporal data and were included in this specific analysis. The median onset time was 79.6 days (IQR: 18.0–251.8 days), showing a right-skewed distribution. To further evaluate the time-varying pattern, a Weibull distribution model was also applied. The shape parameter (β) was calculated at 0.74 (95% CI: 0.68–0.79), mathematically classifying the overall hazard function as an “early failure” type, indicating that the highest instantaneous hazard rate of AEs occurs early in the therapy.

### 3.2 Signals of SOC

**FIGURE 4:**
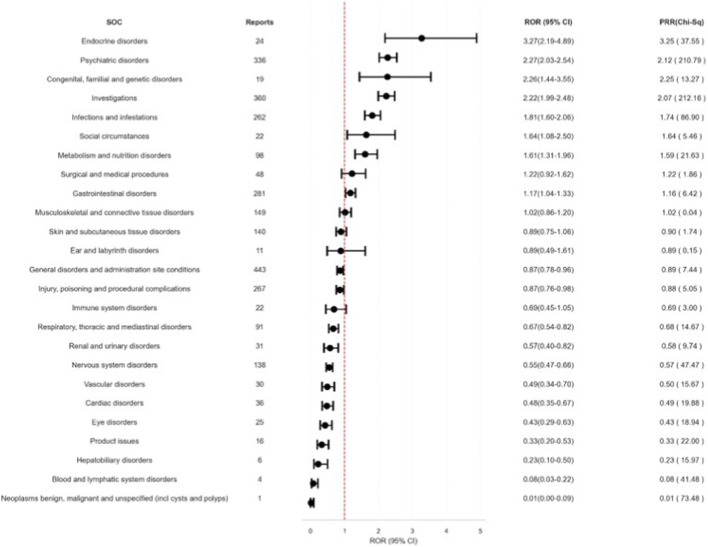
Disproportionality signals of Vamorolone at the SOC level. Note: To provide a comprehensive overview of the baseline data, the overall SOC distribution of all unfiltered records involving Vamorolone is presented in this Figure 4, reflecting the initial raw reporting landscape.

Figure 4 illustrates the overall disproportionality signals of Vamorolone-associated adverse events categorized by SOC. The most prominent signal was observed in Endocrine disorders (ROR = 3.27, 95% CI: 2.19–4.89). Furthermore, Psychiatric disorders (n = 336; ROR = 2.27) and Investigations (n = 360; ROR = 2.22) emerged as major safety concerns, characterized by robust disproportionality metrics and substantial case volumes. A strong signal was also noted in Congenital, familial and genetic disorders (ROR = 2.26). Other categories, such as Infections and infestations (ROR = 1.81) and Metabolism and nutrition disorders (ROR = 1.61), similarly demonstrated significant disproportionality. To uncover specific safety signals that might be obscured at the broader SOC level, a more granular analysis was subsequently conducted at the PT level.

### 3.3 Signals of PT

**FIGURE 5:**
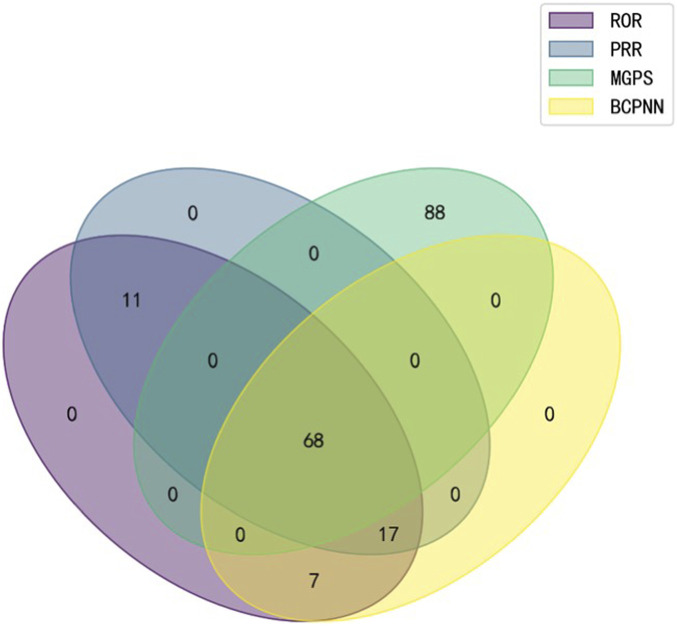
Overlap of positive safety signals detected by the ROR, PRR, BCPNN, and MGPS algorithms.

Based on the four-algorithm consensus approach, an initial set of 68 significant adverse event signals was detected at the PT level. To ensure the clinical relevance of the safety profile and rigorously address confounding by indication, we systematically reviewed and excluded 8 PTs that were explicitly related to the underlying disease progression, patient management, or product/device issues (namely,: *Duchenne muscular dystrophy, wheelchair user, walking disability, gastrostomy, central venous catheterisation, product use complaint, product dose omission issue,* and *syringe issue*). Consequently, a final refined cohort of 60 potential adverse event signals was identified (Figure 5), which are comprehensively detailed in Supplementary Table S2. Table 1 summarizes the top 30 Vamorolone-associated AE signals at the PT level, ranked by their EBGM values. The refined safety profile encompasses both anticipated glucocorticoid-like effects and clinically meaningful unexpected signals. Consistent with the known safety profile, psychiatric disturbances were notably prevalent and yielded the highest disproportionality scores, spearheaded by Stubbornness (n = 3; EBGM = 163.78), Behaviour disorder (n = 25; EBGM = 106.65), and Anger (n = 32; EBGM = 20.18). In the endocrine and metabolic domains, known risks including acute adrenocortical insufficiency (n = 6; EBGM = 71.14), Cushingoid (n = 10; EBGM = 69.04), Increased appetite (n = 52; EBGM = 62.47), and Weight increased—which recorded the highest overall case volume (n = 186; EBGM = 18.31)—were robustly detected. Importantly, our analysis highlighted several potentially novel and unexpected safety signals that warrant high clinical vigilance. For instance, severe infectious complications such as Skin bacterial infection (n = 3; EBGM = 84.11) and Gastroenteritis viral (n = 34; EBGM = 41.03) emerged as prominent unlabelled findings. Other notable unexpected observations included Enuresis (n = 8; EBGM = 42.40) and cardiac markers such as Troponin I increased (n = 3; EBGM = 39.54).

**TABLE 1:** Top 30 Vamorolone-associated adverse event signals at the PT level, sorted in descending order by EBGM values.

PT	SOC	Cases	ROR (95%Cl)	PRR (χ2)	EBGM(EBGM05)	IC(IC025)
Stubbornness	Psychiatric disorders	3	165.25 (53.03–515.00)	165.08 (485.39)	163.78 (52.55)	7.36 (0.52)
Behaviour disorder	Psychiatric disorders	25	108.14 (72.87–160.48)	107.20 (2,616.94)	106.65 (71.87)	6.74 (3.83)
Skin bacterial infection	Infections and infestations	3	84.54 (27.19–262.88)	84.45 (246.38)	84.11 (27.05)	6.39 (0.50)
Adrenocortical insufficiency acute	Endocrine disorders	6	71.53 (32.07–159.58)	71.39 (414.98)	71.14 (31.89)	6.15 (1.60)
Cushingoid	Endocrine disorders	10	69.51 (37.32–129.46)	69.27 (670.60)	69.04 (37.07)	6.11 (2.39)
Increased appetite	Metabolism and nutrition disorders	52	63.79 (48.47–83.96)	62.65 (3,146.18)	62.47 (47.46)	5.97 (4.45)
Skin striae	Skin and subcutaneous tissue disorders	4	59.49 (22.28–158.85)	59.41 (229.06)	59.24 (22.19)	5.89 (0.93)
Impetigo	Infections and infestations	3	43.69 (14.07–135.72)	43.65 (124.76)	43.56 (14.02)	5.44 (0.46)
Enuresis	Psychiatric disorders	8	42.60 (21.27–85.33)	42.48 (323.42)	42.40 (21.17)	5.41 (1.96)
Gastroenteritis viral	Infections and infestations	34	41.60 (29.65–58.35)	41.11 (1,328.45)	41.03 (29.25)	5.36 (3.77)
Troponin i increased	Investigations	3	39.65 (12.77–123.15)	39.61 (112.69)	39.54 (12.73)	5.31 (0.45)
Atypical pneumonia	Infections and infestations	5	36.71 (15.26–88.33)	36.65 (173.07)	36.58 (15.20)	5.19 (1.22)
Troponin increased	Investigations	8	23.54 (11.75–47.13)	23.47 (171.96)	23.45 (11.71)	4.55 (1.78)
Macule	Skin and subcutaneous tissue disorders	3	23.32 (7.51–72.40)	23.30 (63.96)	23.27 (7.50)	4.54 (0.38)
Anger	Psychiatric disorders	32	20.41 (14.40–28.93)	20.20 (583.59)	20.18 (14.24)	4.33 (3.17)
Pharyngitis streptococcal	Infections and infestations	10	20.05 (10.77–37.32)	19.98 (180.20)	19.97 (10.73)	4.32 (2.00)
Emotional disorder	Psychiatric disorders	28	19.65 (13.54–28.51)	19.47 (490.26)	19.45 (13.40)	4.28 (3.03)
Weight increased	Investigations	186	19.53 (16.83–22.66)	18.33 (3,055.10)	18.31 (15.78)	4.19 (3.85)
Frustration tolerance decreased	Psychiatric disorders	6	16.48 (7.39–36.72)	16.45 (86.99)	16.43 (7.37)	4.04 (1.27)
Autism spectrum disorder	Psychiatric disorders	4	16.08 (6.03–42.90)	16.06 (56.46)	16.05 (6.02)	4.00 (0.71)
Mood altered	Psychiatric disorders	19	15.25 (9.71–23.95)	15.16 (251.14)	15.15 (9.64)	3.92 (2.50)
Abnormal behaviour	Psychiatric disorders	27	14.62 (10.00–21.35)	14.49 (338.99)	14.48 (9.91)	3.86 (2.74)
Hair growth abnormal	Skin and subcutaneous tissue disorders	4	12.54 (4.70–33.44)	12.52 (42.38)	12.51 (4.69)	3.65 (0.63)
Ear infection	Infections and infestations	14	11.64 (6.88–19.68)	11.59 (135.42)	11.58 (6.85)	3.53 (2.02)
Aggression	Psychiatric disorders	27	11.66 (7.98–17.04)	11.56 (260.58)	11.56 (7.91)	3.53 (2.52)
Adrenal insufficiency	Endocrine disorders	6	11.48 (5.15–25.57)	11.46 (57.23)	11.45 (5.14)	3.52 (1.11)
Ligament sprain	Injury, poisoning and procedural complications	5	11.03 (4.59–26.54)	11.02 (45.52)	11.01 (4.58)	3.46 (0.87)
Seasonal allergy	Immune system disorders	7	10.97 (5.22–23.03)	10.94 (63.21)	10.94 (5.21)	3.45 (1.26)
Scoliosis	Musculoskeletal and connective tissue disorders	3	10.44 (3.37–32.41)	10.43 (25.58)	10.43 (3.36)	3.38 (0.19)
Upper limb fracture	Injury, poisoning and procedural complications	9	9.71 (5.05–18.68)	9.68 (70.05)	9.68 (5.03)	3.27 (1.46)

FAERS: The U.S. FDA, adverse event reporting system; Cases: Number of reported cases; PT: preferred term; ROR: reporting odds ratio; 95%CI: 95% Confidence Interval; PRR: proportional reporting ratio; χ2: chi-squared; IC: information component; IC025: Information Component 2.fifth percentile; EBGM: empirical Bayes geometric mean; EBGM05: Empirical Bayes Geometric Mean 5th percentile.

### 3.4 Key PT-SOC hierarchical mapping analysis

**FIGURE 6:**
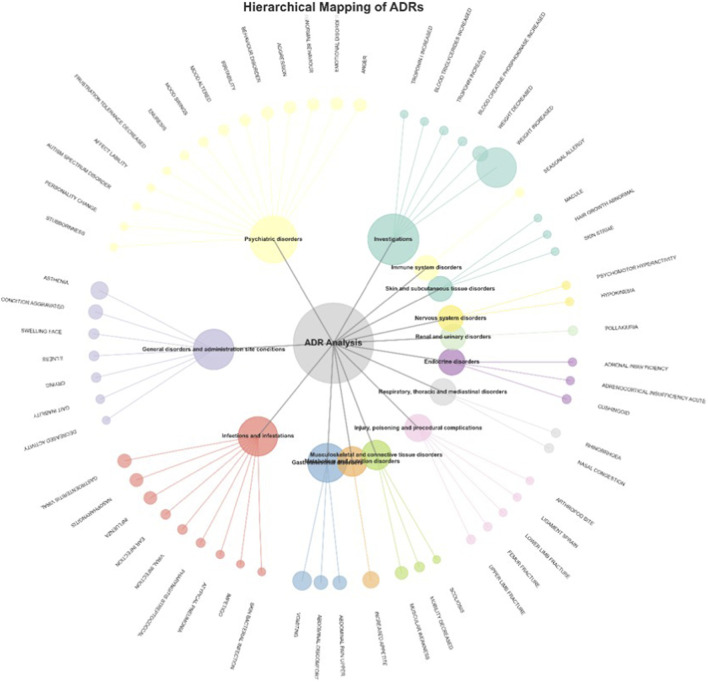
Hierarchical mapping of key adverse event signals to their corresponding SOC.

To contrast with the raw baseline landscape, Figure 6 presents a comprehensive hierarchical mapping of the final 60 positive Vamorolone-associated safety signals, providing a multi-level visualization of validated individual PTs nested within their parent SOCs. The radial distribution effectively captures the refined, multi-systemic safety profile of the drug. The most prominent clusters are concentrated in the Psychiatric disorders (e.g., Abnormal behaviour, Aggression, and Stubbornness), Investigations (e.g., Weight increased and Blood creatine phosphokinase increased), and Infections and infestations (e.g., Ear infection and Gastroenteritis viral) domains.

Furthermore, the mapping reveals a targeted impact across several other crucial physiological systems. This includes notable signals in Gastrointestinal disorders (e.g., Vomiting and Abdominal pain upper), Injury, poisoning and procedural complications (e.g., Femur fracture and Ligament sprain), and General disorders and administration site conditions (e.g., Asthenia and Swelling face). Specific clinical manifestations are also clearly delineated within Metabolism and nutrition disorders (e.g., Increased appetite), Endocrine disorders (e.g., Cushingoid and Adrenocortical insufficiency acute), and Skin and subcutaneous tissue disorders (e.g., Skin striae and Macule). This holistic overview emphasizes the specific clinical landscape of Vamorolone therapy, successfully distilling the complex reporting data into its core psychiatric, metabolic, infectious, and systemic axes.

### 3.5 The spectrum of psychiatric manifestations associated with Vamorolone

**FIGURE 7:**
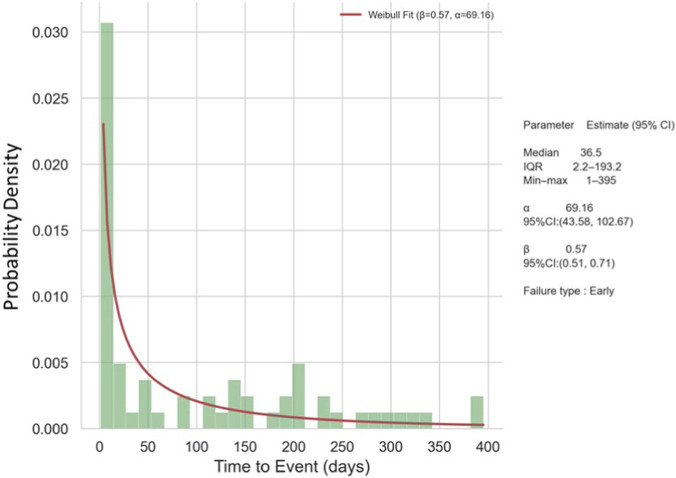
Time-to-onset and weibull distribution analysis of psychiatric safety signals associated with Vamorolone. Note: Time-to-Event: Represents the time interval (in days) from the initiation of therapy to the first occurrence of the adverse event. Probability Density: Reflects the relative temporal likelihood or concentration of event onset, rather than the true clinical incidence rate.

Table 2 provides a detailed breakdown of the key psychiatric safety signals identified for Vamorolone at the PT level. The analysis revealed a diverse spectrum of behavioral and emotional disturbances with significant disproportionate reporting. Among these, Stubbornness exhibited the highest signal intensity (EBGM = 163.78), followed closely by Behaviour disorder (EBGM = 106.65). Several terms related to emotional dysregulation also showed robust signals, including Anger (EBGM = 20.18), Emotional disorder (EBGM = 19.45), Aggression (EBGM = 11.56). Furthermore, signals for Mood swings (EBGM = 8.75) and Irritability (EBGM = 7.50) were clearly identified. The robust signal intensities across these 12 psychiatric PTs underscore the consistent nature of these psychiatric manifestations in the Vamorolone reporting profile. Figure 7 illustrates the TTO profile and Weibull distribution for psychiatric signals associated with Vamorolone. The median onset was 36.5 days (IQR: 2.2–193.2 days). Weibull shape analysis yielded a *β* of 0.57 (95% CI: 0.51, 0.71), mathematically classifying the hazard function as an “early failure” type. This indicates that the highest propensity for psychiatric manifestations occurs shortly after treatment initiation, with the instantaneous hazard rate declining thereafter.

**TABLE 2:** Key psychiatric adverse event signals associated with Vamorolone at the PT level.

PT	Cases	ROR (95%Cl)	PRR (χ2)	EBGM(EBGM05)	IC(IC025)
Stubbornness	3	165.25 (53.03–515.00)	165.08 (485.39)	163.78 (52.55)	7.36 (0.52)
Behaviour disorder	25	108.14 (72.87–160.48)	107.20 (2,616.94)	106.65 (71.87)	6.74 (3.83)
Enuresis	8	42.60 (21.27–85.33)	42.48 (323.42)	42.40 (21.17)	5.41 (1.96)
Anger	32	20.41 (14.40–28.93)	20.20 (583.59)	20.18 (14.24)	4.33 (3.17)
Emotional disorder	28	19.65 (13.54–28.51)	19.47 (490.26)	19.45 (13.40)	4.28 (3.03)
Frustration tolerance decreased	6	16.48 (7.39–36.72)	16.45 (86.99)	16.43 (7.37)	4.04 (1.27)
Autism spectrum disorder	4	16.08 (6.03–42.90)	16.06 (56.46)	16.05 (6.02)	4.00 (0.71)
Mood altered	19	15.25 (9.71–23.95)	15.16 (251.14)	15.15 (9.64)	3.92 (2.50)
Abnormal behaviour	27	14.62 (10.00–21.35)	14.49 (338.99)	14.48 (9.91)	3.86 (2.74)
Aggression	27	11.66 (7.98–17.04)	11.56 (260.58)	11.56 (7.91)	3.53 (2.52)
Mood swings	13	8.78 (5.09–15.15)	8.75 (89.22)	8.75 (5.07)	3.13 (1.72)
Irritability	21	7.55 (4.91–11.59)	7.50 (118.36)	7.50 (4.88)	2.91 (1.91)

FAERS: The U.S. FDA, adverse event reporting system; Cases: Number of reported cases; PT: preferred term; ROR: reporting odds ratio; 95%CI: 95% Confidence Interval; PRR: proportional reporting ratio; χ2: chi-squared; IC: information component; IC025: Information Component 2.fifth percentile; EBGM: empirical Bayes geometric mean; EBGM05: Empirical Bayes Geometric Mean fifth percentile.

### 3.6 Comparative safety profiling of Vamorolone versus Deflazacort across growth, skeletal, endocrine, and psychiatric domains

Tables 3, 4 present a comparative disproportionality analysis between Vamorolone and Deflazacort across growth, skeletal, endocrine, and psychiatric domains. A potentially lower reporting frequency for Vamorolone was observed in domains related to skeletal health and growth preservation. Specifically, Vamorolone exhibited a markedly lower reporting intensity for osteoporosis (Crude OR = 0.27, 95%CI 0.08–0.87, *P* = 0.02) and femur fractures (Crude OR = 0.38, 95%CI 0.18–0.80, *P* = 0.008) compared to Deflazacort. In the endocrine and growth sectors, the signal for growth retardation was significantly reduced (Crude OR = 0.15, 95%CI 0.04–0.63, *P* = 0.003), alongside a lower frequency of Cushingoid features (Crude OR = 0.33, 95%CI 0.17–0.64, *P* < 0.001). However, no significant differences were found for spinal or compression fractures. Similarly, the psychiatric profile—including aggression, anger, and emotional disorders—remained comparable between the two drugs, with no statistically significant differences detected across all psychiatric PTs (*P* > 0.05).

**TABLE 3:** Comparative signal evaluation of growth, skeletal, and metabolic adverse events between Vamorolone and Deflazacort.

PT	Vamorolone (total cases: 2,860)		Deflazacort (total cases: 7,102)		Crude OR (95%CI)	*P*
	Cases(a)	Non-cases(b)	Cases(c)	Non-cases(d)		
Growth retardation	2	2,858	33	7,069	0.15 (0.04–0.63)	**0.003**
Cushingoid	10	2,850	75	7,027	0.33 (0.17–0.64)	**<0.001**
Osteoporosis	3	2,857	28	7,074	0.27 (0.08–0.87)	**0.02**
Femur fracture	8	2,852	52	7,050	0.38 (0.18–0.80)	**0.008**
Compression fracture	2	2,858	6	7,096	0.83 (0.08–4.63)	1.00
Spinal fracture	3	2,857	15	7,087	0.50 (0.14–1.72)	0.26

CI: confidence interval; Crude OR: Odds Ratio calculated directly from a 2 × 2 contingency table (Vamorolone vs. Deflazacort). *P*: The P-value for the Crude OR, was determined using the Pearson chi-square test, or Fisher’s exact test when expected cell counts were less than 5. A Crude OR < 1 with *P* < 0.05 indicates significantly lower reporting odds for Vamorolone compared to deflazacort.

**TABLE 4:** Comparative signal evaluation of psychiatric adverse events between Vamorolone and Deflazacort.

PT	Vamorolone (total cases: 2,860)		Deflazacort (total cases: 7,102)		Crude OR (95%CI)	*P*
	Cases(a)	Non-cases(b)	Cases(c)	Non-cases(d)		
Aggression	27	2,833	71	7,031	0.94 (0.60–1.47)	0.80
Irritability	21	2,839	63	7,039	0.83 (0.50–1.36)	0.45
Anger	32	2,828	97	7,005	0.82 (0.55–1.22)	0.32
Emotional disorder	28	2,832	54	7,048	1.29 (0.82–2.04)	0.27
Mood altered	19	2,841	61	7,041	0.77 (0.46–1.29)	0.33
Mood swings	13	2,847	38	7,064	0.85 (0.45–1.60)	0.61
Depression	8	2,852	31	7,071	0.64 (0.29–1.39)	0.26

CI: confidence interval; Crude OR: Odds Ratio calculated directly from a 2 × 2 contingency table (Vamorolone vs. Deflazacort). *P*: The P-value for the Crude OR, was determined using the Pearson chi-square test, or Fisher’s exact test when expected cell counts were less than 5. A Crude OR < 1 with *P* < 0.05 indicates significantly lower reporting odds for Vamorolone compared to deflazacort.

### 3.7 Multiple-testing robustness assessment

To confirm the internal statistical validity of our findings, a Bonferroni-corrected robustness assessment was performed (Supplementary Table S3). Given the total number of unique Vamorolone-associated PTs tested in our cohort (N = 579), the threshold for statistical significance was adjusted to *P* < 8.636 × 10^−5^. Most of our key identified safety signals—including weight increased, product dose omission issue, vomiting, and specific psychiatric or endocrine manifestations—remained statistically significant after this correction. This indicates that our primary findings are robust and are unlikely to be false positives resulting from multiple testing.

## 4 Discussion

As a novel dissociative corticosteroid indicated for DMD, Vamorolone is engineered to decouple therapeutic efficacy from typical glucocorticoid toxicities through three distinct structure-activity modifications compared to conventional agents like prednisone and deflazacort. First, it bypasses the typical pro-drug conversion pathways, as it is not metabolized by 11*β*-hydroxysteroid dehydrogenase types 1 and 2 (11*β*-HSD1/2) (Crastin et al., 2025; Hoffman et al., 2018). Second, Vamorolone exhibits an atypical binding profile with the glucocorticoid receptor, characterized by diminished co-activator recruitment coupled with enhanced co-repressor affinity relative to classic steroids (Liu et al., 2020). Finally, its inherent activity as a mineralocorticoid receptor antagonist theoretically offers supplementary cardiovascular protection, a potential benefit that warrants further clinical exploration (Heier et al., 2019; de Vera et al., 2025). However, whether these sophisticated molecular designs fully translate into a safer clinical profile in broader, real-world populations requires robust epidemiological validation. Based on the expansive data repository of FAERS, this study provides the first comprehensive and systematic characterization of real-world post-marketing safety signals for Vamorolone. Analysis of the 1,171 adverse event reports where Vamorolone was identified as the primary suspected medication revealed that the detected signals were distributed across multiple SOCs, with the highest concentrations in the endocrine, psychiatric, and investigation categories. Macroscopically, our findings largely validate the initial design objectives and advantages of Vamorolone in specific domains such as skeletal health and growth preservation. However, the distribution of signal intensities at the SOC level indicates that endocrine disorders (ROR = 3.27), psychiatric disorders (ROR = 2.27), and various investigation abnormalities primarily involving weight and metabolic parameters (ROR = 2.22) constitute the most prominent safety challenges in real-world clinical practice.

Although no adverse events related to adrenal insufficiency were reported in the two key randomized controlled trials of Vamorolone (Dang et al., 2024; Gugl et al., 2022), the present study identified exceptionally strong signals for acute adrenocortical insufficiency (EBGM = 71.14) and Cushingoid features (EBGM = 69.04) in the endocrine domain. These findings suggest that Vamorolone’s “dissociative” design does not exempt it from inducing profound hypothalamic-pituitary-adrenal (HPA) axis suppression. Mechanistically, as an exogenous glucocorticoid, Vamorolone-bound glucocorticoid receptors retain transrepression activity and can negatively feedback to inhibit the synthesis and secretion of hypothalamic CRH as well as pituitary POMC/ACTH, thereby suppressing endogenous cortisol production and ultimately resulting in adrenal insufficiency (Liu et al., 2020; Ahmet et al., 2025; Gjerstad et al., 2018; Grounds and Lloyd, 2023; D'Ambrosio and Mendell, 2023). Our real-world observations are strongly corroborated by *post hoc* analyses of the VISION-DMD trial (Ahmet et al., 2025). Clinical data revealed dose-dependent adrenal suppression (AS) in nearly all subjects, with AS rates reaching 95.2% in the high-dose group (6 mg/kg/day) at week 24 when using the 500 nmol/L threshold (Ahmet et al., 2025). Even with a more stringent threshold (<400 nmol/L) optimized for modern immunoassays, the AS rate remained remarkably high at 90.5% (Ahmet et al., 2025). These consistent results underscore the necessity for rigorous adrenal monitoring and stress-dose protocols in patients transitioning to or maintained on Vamorolone. Moreover, recent analyses underscore the pervasive withdrawal risks of traditional corticosteroids (Mangal et al., 2025). Similarly, our findings emphasize that novel dissociative steroids like Vamorolone still necessitate strict vigilance for related endocrine disturbances, particularly HPA axis suppression. Therefore, given the acute adrenocortical insufficiency identified in this study, current clinical guidelines recommend a standardized target dose of 6 mg/kg/day for patients switching from any previous corticosteroid therapy to Vamorolone in order to avoid acute adrenal crisis (Ahmet et al., 2025).

Regarding the psychiatric domain, this study identified an extensive range of significant safety signals, including stubbornness (EBGM = 163.78), behaviour disorder (EBGM = 106.65), enuresis (EBGM = 42.40), anger (EBGM = 20.18), and aggression (EBGM = 11.56). It is important to note that disproportionality analysis solely identifies reporting associations and cannot establish biological causality. However, existing preclinical literature provides a speculative pharmacological context for these observations. Studies using mdx DMD mouse models have demonstrated that traditional glucocorticoids, such as prednisolone and deflazacort, are strong substrates for P-glycoprotein (P-gp) (Karssen et al., 2002). As a key efflux pump at the blood-brain barrier (BBB), P-gp limits the CNS accumulation of these traditional steroids. In contrast, Vamorolone is not a high-affinity P-gp substrate, which theoretically allows it to achieve higher drug exposure in the brain (Liu et al., 2024). Once within the CNS, Vamorolone has been shown to act on glucocorticoid receptors to induce widespread transcriptomic changes, which have been linked to depressive-like behaviors in animal models—effects notably more pronounced than those observed with deflazacort (Liu et al., 2024; Zhao et al., 2008; Kajiyama et al., 2010). The high reporting intensities for externalizing behaviors such as stubbornness, anger, and behavioral disorders observed in our FAERS analysis may align with these preclinical hypotheses regarding CNS exposure, though a direct mechanistic link cannot be confirmed from our data. Pediatric DMD patients are especially sensitive to neurochemical fluctuations during development, where an exogenous receptor agonist might theoretically disrupt the balance between the frontal lobe and limbic system. Consequently, given the observed reporting signals and the theoretical background of BBB permeability, vigilant psychiatric monitoring and consistent oversight of emotional patterns in children are warranted.

Despite the endocrine and psychiatric signals identified, this study provides exploratory real-world evidence that aligns with the therapeutic intent of Vamorolone, generating the hypothesis that it may mitigate growth and skeletal toxicities while maintaining anti-inflammatory efficacy. In the clinical management of DMD, chronic use of traditional corticosteroids is frequently associated with severe growth retardation and osteoporosis, which can accelerate the loss of ambulation (Bello et al., 2015; Griggs et al., 2013; Herbelet et al., 2020). These toxicities are primarily attributed to the transactivation pathway, which promotes osteoblast apoptosis and impairs bone formation (Herbelet et al., 2020). In the context of Vamorolone, 11*β*-hydroxysteroid dehydrogenase type 1 (11*β*-HSD1) plays a critical role in this process by converting inactive glucocorticoids into their active forms locally, thereby mediating the suppression of anabolic bone formation and the reduction in osteoblast numbers (Fenton et al., 2019). Our comparative analysis reflects the clinical manifestation of this pharmacological distinction. Compared to deflazacort, Vamorolone was associated with a lower reporting frequency across several growth and bone-related domains: growth retardation, osteoporosis, femur fracture. These observations from the FAERS database are consistent with clinical trial data and bone turnover biomarker studies (Dang et al., 2024). Prior research has indicated that transitioning to Vamorolone can stabilize bone formation markers and, in some cases, support a recovery toward normal growth percentiles (Dang et al., 2024; Gugl et al., 2022; Mah et al., 2022). While no significant difference was observed for spinal or compression fractures—potentially due to the inherent biomechanical progression of advanced DMD—the overall reporting patterns in this study suggest a more favorable skeletal and growth safety profile for Vamorolone in a real-world setting.

Finally, this study utilized the Weibull distribution model to explore the onset patterns of Vamorolone-associated adverse events. The median TTO for overall AEs was 79.6 days, with a fitted shape parameter (*β*) of 0.74 (95% CI: 0.68–0.79). Notably, when psychiatric signals were analyzed independently, the median TTO shortened to 36.5 days, with a *β* of 0.57 (95% CI: 0.51–0.71). Since these *β* values are significantly less than 1, Vamorolone’s safety signals are characterized as an “early failure” type. While the declining hazard rate over time may reflect physiological adaptation or the “depletion of susceptibles,” the intrinsic nature of Vamorolone as a potent steroidal ligand implies that cumulative exposure risks remain. Clinical management must therefore balance the vigilance required for acute early-stage reactions with the sustained monitoring necessary to address the insidious, chronic systemic effects common to long-term glucocorticoid receptor modulation.

In summary, this real-world pharmacovigilance analysis comprehensively delineates the nuanced risk-benefit profile of Vamorolone. While the comparative reporting data suggests potential skeletal and growth-sparing reporting advantages over traditional glucocorticoids, it is not exempt from severe systemic toxicities. The pronounced early-onset psychiatric disturbances—driven by its high CNS penetrance—and notable HPA axis suppression underscore the drug’s potent steroidal nature. Ultimately, optimizing Vamorolone therapy in DMD requires clinicians to leverage its bone-protective benefits while implementing proactive, rigorous monitoring for adrenal and neurobehavioral adverse events, particularly during the critical early phases of treatment.

## 5 Strengths and limitations

This study provides the first comprehensive real-world pharmacovigilance assessment of the novel dissociative steroid Vamorolone by leveraging the expansive FAERS database, employing four complementary signal detection algorithms, and utilizing a direct head-to-head contingency analysis against the traditional gold-standard deflazacort. Despite these strengths, our findings must be interpreted in light of several limitations inherent to spontaneous reporting systems.

First, FAERS data are subject to inherent biases such as underreporting and incomplete clinical records. Important covariates—including age, sex, dose, treatment duration, disease severity, reporting year, reporter type, and prior or concomitant corticosteroid exposure—were incompletely captured or not adjusted for in the present analysis. These factors may have influenced reporting patterns and the between-drug comparative ORs. Therefore, the detected signals should be interpreted strictly as differential reporting associations rather than direct estimates of absolute clinical risk. Second, because Vamorolone was only recently approved by the FDA in late 2023, the current safety profile may be influenced by the Weber effect—a recognized epidemiological phenomenon where adverse event reporting artificially peaks shortly after a new drug enters the market. Third, although we systematically reviewed and excluded explicit artifactual signals related to the specific indication (e.g., Duchenne muscular dystrophy), spontaneous reports remain inherently susceptible to residual confounding by indication. Furthermore, while a conservative Bonferroni correction was applied to assess statistical robustness against multiple testing, a conventional sensitivity analysis evaluating alternative case definitions was not feasible due to the risk of massive polypharmacy confounding; thus, prolonged exposure to traditional corticosteroids cannot be entirely ruled out. Fourth, the “early failure type” onset pattern identified via the Weibull distribution model may be partially biased by stringent early-treatment monitoring protocols and reporting delays, warranting cautious clinical interpretation. Finally, our findings are derived exclusively from the FAERS database. Future studies utilizing independent international databases (e.g., WHO VigiBase or EudraVigilance) and prospective clinical cohorts are warranted to externally validate these hypothesis-generating signals and enhance the global generalizability of the comparative safety profile.

## 6 Conclusion

In conclusion, this real-world disproportionality analysis provides evidence that strongly aligns with the anticipated clinical benefits of Vamorolone’s dissociative molecular design, generating the hypothesis that Vamorolone may possess a differentiated, potentially more favorable safety reporting profile regarding skeletal health and growth preservation compared to traditional glucocorticoids like deflazacort. However, its clinical application is not exempt from severe systemic toxicities. The robust signals for acute adrenocortical insufficiency and early-onset psychiatric disturbances are consistent with HPA axis suppression and the drug’s high central nervous system penetrance. Therefore, optimizing Vamorolone therapy in pediatric DMD patients requires a nuanced clinical approach: practitioners must leverage its indicated bone-protective advantages while implementing proactive, rigorous monitoring for adrenal and neurobehavioral adverse events, particularly during the critical first 3 months of treatment initiation.

## Data Availability

The datasets presented in this study can be found in online repositories. The names of the repository/repositories and accession number(s) can be found below: Publicly available datasets were analyzed in this study. These data can be found here: https://fis.fda.gov/extensions/FPD-QDE-FAERS/FPD-QDE-FAERS.html.
