# Supplementary material for: Post-marketing safety surveillance and signal characterization of the novel dissociative steroid Vamorolone in Duchenne muscular dystrophy: a comparative disproportionality analysis based on FAERS data
Source: Front Pharmacol. 2026 Jun 26;17:1838974. doi: 10.3389/fphar.2026.1838974 (PMC13350262; doi:10.3389/fphar.2026.1838974)
Supplement: Supplementary file 1 [file Table1.docx]

# The READUS-PV Checklist

| **Section and topic** | **Item** | **Checklist item** | **Location where item is reported** |
| --- | --- | --- | --- |
| Title | 1a | If disproportionality analyses are a prominent component of the published study, the study should be identified as a “disproportionality analysis”. The type of data and name of the database(s) should be specified. | Title, Abstract, Methods (2.1, 2.3); Explicitly identified as a 'comparative disproportionality analysis' based on FAERS data. |
|  | 1b | Report the name of adverse event(s) and/or drug(s) under study, when applicable. | Title, Abstract, Introduction; Drug: Vamorolone; Adverse events: Post-marketing safety signals across endocrine, psychiatric, skeletal, and growth domains. |
| Introduction Background | 2a | Describe the drug(s) and its utilization, the nature of the adverse event(s) under study and its frequency, and the existing knowledge on the drug-event combination. | Introduction (Paragraphs 1-3); Describes DMD, the severe toxicities of traditional glucocorticoids, and introduces Vamorolone as a novel dissociative steroid designed to attenuate these toxicities. |
|  | 2b | Specify the rationale for performing the analysis, e.g., as part of routine pharmacovigilance, to investigate an overall safety profile, or to assess a pre-specified hypothesis. | Introduction (Paragraphs 4-5); Aims to systematically evaluate Vamorolone's real-world safety profile and conduct a comparative analysis against traditional therapies (deflazacort). |
|  | 2c | Explain why ICSR databases and disproportionality analysis are suitable to fill the knowledge gap. | Introduction (Paragraph 4); Highlights that clinical trials have inherent limitations (restricted sample sizes, limited duration), making large-scale FAERS data crucial for evaluating post-marketing safety. |
| Objectives | 3 | State specific objectives, identifying the adverse event(s), the drug(s), and the reference group, including any pre-specified hypothesis, if applicable. | Abstract (Objective), Introduction, Methods (2.3); Target drug: Vamorolone; Reference group: FAERS database background (for overall disproportionality) and Deflazacort (for direct head-to-head 2x2 comparative analysis); Objective: To comprehensively evaluate the real-world safety profile and compare it with traditional glucocorticoids. |
| Methods Study design | 4a | Describe the study design (i.e., disproportionality analysis) and the type of data used (i.e., spontaneous adverse event reports). | Methods (2.1, 2.3); Study type: Disproportionality analysis; Data type: Spontaneous adverse event reports from FAERS. |
|  | 4b | Provide an outline of the entire study design, including primary and sensitivity analyses performed, and other designs such as case-by-case analysis or literature review. | Methods (2.2, 2.3); Outline: Data extraction (2023Q4–2025Q4) -> Deduplication -> Disproportionality (ROR, PRR, BCPNN, EBGM) -> Weibull time-to-onset analysis -> Comparative analysis (Direct OR from 2x2 tables) -> Sensitivity analysis (monotherapy). |
| Data source | 5a | Describe the ICSR database(s) used (e.g., population coverage, source of reports) and its/their custodian(s). | Methods (2.1, 2.2); Database: FAERS; Custodian: U.S. FDA; Coverage: Global spontaneous reports encompassing 7 distinct data files. |
|  | 5b | Specify the period from which data were extracted, the date of data access/extraction, and any processing (e.g., quality control/data cleaning/duplicate management) applied to the dataset. | Methods (2.1, 2.2); Extraction period: 2023Q4 to 2025Q4. Deduplication was performed globally using FDA-recommended algorithm (latest FDA_DT and largest PRIMARY_ID) prior to target extraction. |
| Variables definition | 6a | Define the study population (e.g., all reports in the database or a subset). | Methods (2.2); Study population: All cases in FAERS where Vamorolone (or Deflazacort for comparison) was identified as the Primary Suspect (PS) drug. |
|  | 6b | Define all variables of interest (e.g., age, sex, setting) and their coding. | Methods (2.2, 2.3); Key variables: Demographics (age, sex, weight), reporting countries, reporter types, severity outcomes, and time-to-onset (TTO). |
|  | 6c | Specify and justify any grouping of drugs or events. For drugs, specify and justify whether active ingredients/trade names/salts were considered and/or the selected role. | Methods (2.1, 2.2); Drugs were identified using generic ('VAMOROLONE') and trade names ('AGAMREE'). Events were grouped using MedDRA Preferred Terms (PTs) and System Organ Classes (SOCs). |
|  | 6d | If denominators are used to estimate reporting rates (e.g., number of prescriptions), specify their definition and data source. | Not applicable. No external denominators (like prescription volume) were used to calculate incidence rates. |
| Analysis | 7a | Describe descriptive analysis performed. | Methods (2.3), Results (3.1); Descriptive statistics for demographics, clinical characteristics, and Weibull distribution for time-to-onset (TTO). |
|  | 7b | Describe disproportionality analysis performed. | Methods (2.3); Algorithms: ROR, PRR, BCPNN, EBGM. Signal thresholds defined (e.g., N>=3, EBGM05>2). Direct 2x2 OR calculated for comparative safety analysis with Fisher/Chi-square tests for P-values. |
|  | 7c | Describe sensitivity analyses performed. | Methods (2.3); Sensitivity analysis strictly filtered for monotherapy to minimize immediate drug-drug interactions. |
|  | 7d | Describe case-by-case evaluation/causality assessment performed. | Not applicable. No individual case causality assessment was conducted. |
|  | 7e | Describe other specific analyses performed (e.g., literature review). | Not applicable. No other specific analyses performed. |
| Results Study population | 8a | Report the total number of reports and/or drug-event combinations resulting from data extraction and processing, giving reasons for exclusions at each stage (e.g., via a flow chart). | Results (3.1), Figure 1; Total database preprocessing yielded millions of records, refining down to 1,171 Vamorolone cases and 2,860 PT-level AEs. |
|  | 8b | Describe the characteristics of the study population (e.g., distribution by age, sex, or other factors). | Results (3.1), Figure 2; 97.0% male, 53.0% pediatric (2-17 years), 99.0% from the US. |
| Main results | 9 | Present all results including confidence intervals. Present also results of sensitivity analyses, if performed. | Results (3.2-3.7); Presented SOC RORs, Top 30 PTs with EBGM values, Weibull beta values (overall and psychiatric), and comparative Direct ORs and P-values from 2x2 contingency tables. Signals were cross-referenced with labeling to classify known vs. unexpected safety signals. |
| Case-by-case evaluation | 10 | Report results from case-by-case evaluation/causality assessment, if performed. | Not applicable. |
| Discussion Interpretation of results | 11 | Discuss key results with reference to study objectives and existing knowledge, keeping in mind the exploratory nature of the analyses. Suggest potential causal mechanisms when appropriate. | Discussion (All paragraphs); Correlates Vamorolone's bone-sparing Direct OR advantages with clinical trials. Discusses known risks (adrenal suppression) and unexpected hypothesis-generating signals (e.g., Troponin I increased). Warns of careful interpretation of reporting patterns. |
| Generalizability and clinical implications | 12a | Discuss generalizability (external validity) of the results. | Results (3.1), Discussion; Characteristics of the cohort (primarily pediatric males with DMD in the US) limit generalizability to other populations. |
|  | 12b | Discuss the clinical implications of the results. | Discussion, Conclusion; Recommends adherence to 6 mg/kg/day transition dose to avoid adrenal crisis and vigilant psychiatric monitoring in the first 3 months. Emphasizes external validation for novel signals. |
|  | 12c | Discuss the subsequent study designs, if recommended. | Strengths and limitations (Section 5); Recommends utilizing independent international databases (VigiBase, EudraVigilance) and prospective clinical cohorts for external validation. |
| Limitations | 13 | Present general limitations, making clear that disproportionality analysis alone cannot prove causation or measure incidence, and specific limitations, including confounding and reporting bias and efforts to mitigate them. | Strengths and limitations (Section 5); Acknowledges FAERS biases (underreporting, confounding by indication), the Weber effect, and explicitly states that signals are 'differential reporting associations' rather than direct risk estimates. |
| Declarations | 14a | Provide the source of funding/sponsorship and the role of the funders/sponsors for the present study and for any original study on which the present article is based. | Declarations (Funding); Acknowledges funding sources. Role of funder not explicitly stated. |
|  | 14b | Clearly identify potential commercial and intellectual conflicts of interest. | Declarations (Conflict of interest); The authors declare no competing or commercial interests. |
|  | 14c | Declare any institutional approval needed or granted in the investigation. | Declarations (Ethical approval); Not applicable as data is fully de-identified and publicly available. |
|  | 14d | Include a statement on data availability, code availability, and protocol registration. | Declarations (Data availability); FAERS data is publicly available. Software (MySQL, R, Excel) listed in Methods. |
