# Supplementary material for: Post-marketing safety surveillance and signal characterization of the novel dissociative steroid Vamorolone in Duchenne muscular dystrophy: a comparative disproportionality analysis based on FAERS data
Source: Front Pharmacol. 2026 Jun 26;17:1838974. doi: 10.3389/fphar.2026.1838974 (PMC13350262; doi:10.3389/fphar.2026.1838974)
Supplement: Supplementary file 2 [file Table2.docx]

**Supplementary Table**

**Table S1 A two-by-two contingency table and detailed formulas for disproportionality analysis**

|  | Vamorolone-related AEs | Other AEs | | Sums |
| --- | --- | --- | --- | --- |
| Vamorolone | a | | b | a+b |
| Other drugs | c | | d | c+d |
| Sums | a+c | | b+d | a+b+c+d |
|  |  | |  |  |
| Algorithms | Calculation formulas | |  |  |
| ROR | $ROR=\frac{ad}{bc}$ | | | a≥3 and 95% CI (lower limit)>1 |
|  | $95\%CI=e^{ln(ROR)\pm1.96\sqrt{\frac{1}{a}+\frac{1}{b}+\frac{1}{c}+\frac{1}{d}}}$ | | |  |
| PRR | $PRR=\frac{a(c+d)}{c(a+b)}$ | | | a≥3 and PRR ≥ 2 and χ^2^ ≥ 4 |
|  | $95\%CI=e^{ln(PRR)\pm1.96\sqrt{\frac{1}{a}-\frac{1}{a+b}+\frac{1}{c}-\frac{1}{c+d}}}$ | | |  |
|  | $\chi^{2}=\frac{\left[ \left( ad-bc \right)^{2} \right]\left( a+b+c+d \right)}{\left[ \left( a+b \right)\left( c+d \right)\left( a+c \right)\left( b+d \right) \right]}$ | | |  |
| BCPNN | $IC=\log_{2} \frac{a(a+b+c+d)}{\left( a+c \right)\left( a+b \right)}$ | | | IC025>0 |
|  | $E\left( IC \right)=\log_{2} \frac{(a+\gamma11)(a+b+c+d+\alpha)(a+b+c+d+\beta)}{\left( a+b+c+d+\gamma\right)\left( a+b+\alpha1 \right)\left( a+c+\beta1 \right)}$ | | |  |
|  | $V\left( IC \right)=\frac{1}{{(ln2)}^{2}}\{\left[ \frac{\left( a+b+c+d \right)-a+\gamma-\gamma11}{\left( a+\gamma11 \right)\left( 1+a+b+c+d+\gamma\right)} \right]+\left[ \frac{\left( a+b+c+d \right)-\left( a+b \right)+\alpha-\alpha1}{\left( a+b+\alpha1 \right)\left( 1+a+b+c+d+\alpha\right)} \right]+\left[ \frac{\left( a+b+c+d \right)-\left( a+c \right)+\beta-\beta1}{\left( a+c+\beta1 \right)\left( 1+a+b+c+d+\beta\right)} \right]\}$ | | | |
|  | $95\%CI=IC025=E\left( IC \right)-2\sqrt{V(IC)}$ | | |  |
|  | $\gamma=\gamma11\frac{(a+b+c+d+\alpha)(a+b+c+d+\beta)}{(a+b+\alpha1)(a+c+\beta1)}$ | | |  |
| MGPS | $openEBGM package in R$ | | | EBGM05>2 |

Note: EBGM and its confidence intervals (EBGM05, EBGM95) were calculated using the openEBGM package (version 0.9.1) in R

**Table S2 A Signal strength of all adverse event reports for Vamorolone at the PT level in the FAERS database**

| **PT** | **SOC** | **Cases** | **ROR(95%Cl)** | **PRR(χ2)** | **EBGM(EBGM05)** | **IC(IC025)** |
| --- | --- | --- | --- | --- | --- | --- |
| Stubbornness | Psychiatric disorders | 3 | 165.25 ( 53.03 - 515.00 ) | 165.08 ( 485.39 ) | 163.78 ( 52.55) | 7.36 ( 0.52 ) |
| Behaviour disorder | Psychiatric disorders | 25 | 108.14 ( 72.87 - 160.48 ) | 107.20 ( 2616.94 ) | 106.65 ( 71.87) | 6.74 ( 3.83 ) |
| Skin bacterial infection | Infections and infestations | 3 | 84.54 ( 27.19 - 262.88 ) | 84.45 ( 246.38 ) | 84.11 ( 27.05) | 6.39 ( 0.50 ) |
| Adrenocortical insufficiency acute | Endocrine disorders | 6 | 71.53 ( 32.07 - 159.58 ) | 71.39 ( 414.98 ) | 71.14 ( 31.89) | 6.15 ( 1.60 ) |
| Cushingoid | Endocrine disorders | 10 | 69.51 ( 37.32 - 129.46 ) | 69.27 ( 670.60 ) | 69.04 ( 37.07) | 6.11 ( 2.39 ) |
| Increased appetite | Metabolism and nutrition disorders | 52 | 63.79 ( 48.47 - 83.96 ) | 62.65 ( 3146.18 ) | 62.47 ( 47.46) | 5.97 ( 4.45 ) |
| Skin striae | Skin and subcutaneous tissue disorders | 4 | 59.49 ( 22.28 - 158.85 ) | 59.41 ( 229.06 ) | 59.24 ( 22.19) | 5.89 ( 0.93 ) |
| Impetigo | Infections and infestations | 3 | 43.69 ( 14.07 - 135.72 ) | 43.65 ( 124.76 ) | 43.56 ( 14.02) | 5.44 ( 0.46 ) |
| Enuresis | Psychiatric disorders | 8 | 42.60 ( 21.27 - 85.33 ) | 42.48 ( 323.42 ) | 42.40 ( 21.17) | 5.41 ( 1.96 ) |
| Gastroenteritis viral | Infections and infestations | 34 | 41.60 ( 29.65 - 58.35 ) | 41.11 ( 1328.45 ) | 41.03 ( 29.25) | 5.36 ( 3.77 ) |
| Troponin i increased | Investigations | 3 | 39.65 ( 12.77 - 123.15 ) | 39.61 ( 112.69 ) | 39.54 ( 12.73) | 5.31 ( 0.45 ) |
| Atypical pneumonia | Infections and infestations | 5 | 36.71 ( 15.26 - 88.33 ) | 36.65 ( 173.07 ) | 36.58 ( 15.20) | 5.19 ( 1.22 ) |
| Troponin increased | Investigations | 8 | 23.54 ( 11.75 - 47.13 ) | 23.47 ( 171.96 ) | 23.45 ( 11.71) | 4.55 ( 1.78 ) |
| Macule | Skin and subcutaneous tissue disorders | 3 | 23.32 ( 7.51 - 72.40 ) | 23.30 ( 63.96 ) | 23.27 ( 7.50) | 4.54 ( 0.38 ) |
| Anger | Psychiatric disorders | 32 | 20.41 ( 14.40 - 28.93 ) | 20.20 ( 583.59 ) | 20.18 ( 14.24) | 4.33 ( 3.17 ) |
| Pharyngitis streptococcal | Infections and infestations | 10 | 20.05 ( 10.77 - 37.32 ) | 19.98 ( 180.20 ) | 19.97 ( 10.73) | 4.32 ( 2.00 ) |
| Emotional disorder | Psychiatric disorders | 28 | 19.65 ( 13.54 - 28.51 ) | 19.47 ( 490.26 ) | 19.45 ( 13.40) | 4.28 ( 3.03 ) |
| Weight increased | Investigations | 186 | 19.53 ( 16.83 - 22.66 ) | 18.33 ( 3055.10 ) | 18.31 ( 15.78) | 4.19 ( 3.85 ) |
| Frustration tolerance decreased | Psychiatric disorders | 6 | 16.48 ( 7.39 - 36.72 ) | 16.45 ( 86.99 ) | 16.43 ( 7.37) | 4.04 ( 1.27 ) |
| Autism spectrum disorder | Psychiatric disorders | 4 | 16.08 ( 6.03 - 42.90 ) | 16.06 ( 56.46 ) | 16.05 ( 6.02) | 4.00 ( 0.71 ) |
| Mood altered | Psychiatric disorders | 19 | 15.25 ( 9.71 - 23.95 ) | 15.16 ( 251.14 ) | 15.15 ( 9.64) | 3.92 ( 2.50 ) |
| Abnormal behaviour | Psychiatric disorders | 27 | 14.62 ( 10.00 - 21.35 ) | 14.49 ( 338.99 ) | 14.48 ( 9.91) | 3.86 ( 2.74 ) |
| Hair growth abnormal | Skin and subcutaneous tissue disorders | 4 | 12.54 ( 4.70 - 33.44 ) | 12.52 ( 42.38 ) | 12.51 ( 4.69) | 3.65 ( 0.63 ) |
| Ear infection | Infections and infestations | 14 | 11.64 ( 6.88 - 19.68 ) | 11.59 ( 135.42 ) | 11.58 ( 6.85) | 3.53 ( 2.02 ) |
| Aggression | Psychiatric disorders | 27 | 11.66 ( 7.98 - 17.04 ) | 11.56 ( 260.58 ) | 11.56 ( 7.91) | 3.53 ( 2.52 ) |
| Adrenal insufficiency | Endocrine disorders | 6 | 11.48 ( 5.15 - 25.57 ) | 11.46 ( 57.23 ) | 11.45 ( 5.14) | 3.52 ( 1.11 ) |
| Ligament sprain | Injury, poisoning and procedural complications | 5 | 11.03 ( 4.59 - 26.54 ) | 11.02 ( 45.52 ) | 11.01 ( 4.58) | 3.46 ( 0.87 ) |
| Seasonal allergy | Immune system disorders | 7 | 10.97 ( 5.22 - 23.03 ) | 10.94 ( 63.21 ) | 10.94 ( 5.21) | 3.45 ( 1.26 ) |
| Scoliosis | Musculoskeletal and connective tissue disorders | 3 | 10.44 ( 3.37 - 32.41 ) | 10.43 ( 25.58 ) | 10.43 ( 3.36) | 3.38 ( 0.19 ) |
| Upper limb fracture | Injury, poisoning and procedural complications | 9 | 9.71 ( 5.05 - 18.68 ) | 9.68 ( 70.05 ) | 9.68 ( 5.03) | 3.27 ( 1.46 ) |
| Hypokinesia | Nervous system disorders | 6 | 8.95 ( 4.02 - 19.95 ) | 8.94 ( 42.29 ) | 8.93 ( 4.01) | 3.16 ( 0.97 ) |
| Affect lability | Psychiatric disorders | 4 | 8.95 ( 3.35 - 23.86 ) | 8.93 ( 28.18 ) | 8.93 ( 3.35) | 3.16 ( 0.50 ) |
| Arthropod bite | Injury, poisoning and procedural complications | 3 | 8.77 ( 2.82 - 27.20 ) | 8.76 ( 20.61 ) | 8.75 ( 2.82) | 3.13 ( 0.13 ) |
| Mood swings | Psychiatric disorders | 13 | 8.78 ( 5.09 - 15.15 ) | 8.75 ( 89.22 ) | 8.75 ( 5.07) | 3.13 ( 1.72 ) |
| Viral infection | Infections and infestations | 13 | 8.57 ( 4.97 - 14.78 ) | 8.54 ( 86.53 ) | 8.53 ( 4.95) | 3.09 ( 1.70 ) |
| Decreased activity | General disorders and administration site conditions | 4 | 8.16 ( 3.06 - 21.75 ) | 8.15 ( 25.07 ) | 8.14 ( 3.05) | 3.03 ( 0.45 ) |
| Psychomotor hyperactivity | Nervous system disorders | 6 | 7.75 ( 3.48 - 17.26 ) | 7.73 ( 35.16 ) | 7.73 ( 3.47) | 2.95 ( 0.89 ) |
| Crying | General disorders and administration site conditions | 13 | 7.66 ( 4.44 - 13.21 ) | 7.63 ( 74.90 ) | 7.63 ( 4.42) | 2.93 ( 1.60 ) |
| Irritability | Psychiatric disorders | 21 | 7.55 ( 4.91 - 11.59 ) | 7.50 ( 118.36 ) | 7.50 ( 4.88) | 2.91 ( 1.91 ) |
| Personality change | Psychiatric disorders | 3 | 6.89 ( 2.22 - 21.39 ) | 6.89 ( 15.10 ) | 6.89 ( 2.22) | 2.78 ( 0.03 ) |
| Pollakiuria | Renal and urinary disorders | 13 | 6.79 ( 3.94 - 11.71 ) | 6.76 ( 63.87 ) | 6.76 ( 3.92) | 2.76 ( 1.49 ) |
| Blood creatine phosphokinase increased | Investigations | 9 | 6.53 ( 3.39 - 12.56 ) | 6.51 ( 41.97 ) | 6.51 ( 3.38) | 2.70 ( 1.15 ) |
| Influenza | Infections and infestations | 31 | 6.19 ( 4.35 - 8.82 ) | 6.14 ( 133.48 ) | 6.14 ( 4.31) | 2.62 ( 1.89 ) |
| Gait inability | General disorders and administration site conditions | 9 | 6.15 ( 3.20 - 11.83 ) | 6.13 ( 38.69 ) | 6.13 ( 3.19) | 2.62 ( 1.10 ) |
| Blood triglycerides increased | Investigations | 5 | 6.05 ( 2.52 - 14.56 ) | 6.04 ( 21.05 ) | 6.04 ( 2.51) | 2.60 ( 0.54 ) |
| Muscular weakness | Musculoskeletal and connective tissue disorders | 31 | 5.98 ( 4.20 - 8.52 ) | 5.93 ( 127.12 ) | 5.92 ( 4.16) | 2.57 ( 1.85 ) |
| Lower limb fracture | Injury, poisoning and procedural complications | 5 | 5.86 ( 2.44 - 14.08 ) | 5.85 ( 20.09 ) | 5.85 ( 2.43) | 2.55 ( 0.51 ) |
| Swelling face | General disorders and administration site conditions | 17 | 5.70 ( 3.54 - 9.19 ) | 5.68 ( 65.55 ) | 5.68 ( 3.52) | 2.50 ( 1.49 ) |
| Femur fracture | Injury, poisoning and procedural complications | 8 | 5.58 ( 2.79 - 11.17 ) | 5.57 ( 29.99 ) | 5.57 ( 2.78) | 2.48 ( 0.92 ) |
| Mobility decreased | Musculoskeletal and connective tissue disorders | 16 | 4.98 ( 3.05 - 8.15 ) | 4.96 ( 50.67 ) | 4.96 ( 3.04) | 2.31 ( 1.31 ) |
| Abdominal discomfort | Gastrointestinal disorders | 37 | 4.96 ( 3.59 - 6.86 ) | 4.91 ( 115.49 ) | 4.91 ( 3.55) | 2.30 ( 1.68 ) |
| Nasal congestion | Respiratory, thoracic and mediastinal disorders | 12 | 4.52 ( 2.56 - 7.97 ) | 4.50 ( 32.74 ) | 4.50 ( 2.55) | 2.17 ( 1.02 ) |
| Abdominal pain upper | Gastrointestinal disorders | 36 | 3.87 ( 2.79 - 5.38 ) | 3.84 ( 75.78 ) | 3.84 ( 2.76) | 1.94 ( 1.36 ) |
| Nasopharyngitis | Infections and infestations | 32 | 3.83 ( 2.70 - 5.43 ) | 3.80 ( 66.20 ) | 3.80 ( 2.68) | 1.93 ( 1.30 ) |
| Rhinorrhoea | Respiratory, thoracic and mediastinal disorders | 11 | 3.75 ( 2.07 - 6.77 ) | 3.74 ( 22.06 ) | 3.74 ( 2.07) | 1.90 ( 0.77 ) |
| Illness | General disorders and administration site conditions | 15 | 3.45 ( 2.08 - 5.74 ) | 3.44 ( 26.00 ) | 3.44 ( 2.07) | 1.78 ( 0.85 ) |
| Weight decreased | Investigations | 44 | 3.46 ( 2.57 - 4.66 ) | 3.42 ( 75.68 ) | 3.42 ( 2.54) | 1.77 ( 1.26 ) |
| Asthenia | General disorders and administration site conditions | 58 | 3.39 ( 2.61 - 4.40 ) | 3.34 ( 95.69 ) | 3.34 ( 2.58) | 1.74 ( 1.30 ) |
| Vomiting | Gastrointestinal disorders | 66 | 3.12 ( 2.45 - 3.99 ) | 3.08 ( 93.11 ) | 3.07 ( 2.41) | 1.62 ( 1.22 ) |
| Condition aggravated | General disorders and administration site conditions | 40 | 2.96 ( 2.17 - 4.04 ) | 2.93 ( 51.13 ) | 2.93 ( 2.15) | 1.55 ( 1.03 ) |

**FAERS**: The U.S. FDA Adverse Event Reporting System; **Cases**: Number of reported cases; **PT**: Preferred Term; **ROR**: reporting odds ratio; **95%CI**: 95% Confidence Interval; **PRR**: proportional reporting ratio; **χ2**: chi-squared; **IC**: information component; **IC025**: Information Component 2.5th percentile; **EBGM**: empirical Bayes geometric mean; **EBGM05**: Empirical Bayes Geometric Mean 5th percentile

**Table S3 Sensitivity analysis of Vamorolone-associated adverse event signals utilizing the strict Bonferroni correction for multiple testing**

| **PT** | **SOC** | **Cases** | **ROR(95%Cl)** | **PRR(χ2)** | **EBGM(EBGM05)** | **IC(IC025)** | **Bonferroni-*P*** |
| --- | --- | --- | --- | --- | --- | --- | --- |
| Behaviour disorder | Psychiatric disorders | 25 | 108.14 ( 72.87 - 160.48 ) | 107.20 ( 2616.94 ) | 106.65 ( 71.87) | 6.74 ( 3.83 ) | <8.636×10^-5^ |
| Adrenocortical insufficiency acute | Endocrine disorders | 6 | 71.53 ( 32.07 - 159.58 ) | 71.39 ( 414.98 ) | 71.14 ( 31.89) | 6.15 ( 1.60 ) | <8.636×10^-5^ |
| Cushingoid | Endocrine disorders | 10 | 69.51 ( 37.32 - 129.46 ) | 69.27 ( 670.60 ) | 69.04 ( 37.07) | 6.11 ( 2.39 ) | <8.636×10^-5^ |
| Increased appetite | Metabolism and nutrition disorders | 52 | 63.79 ( 48.47 - 83.96 ) | 62.65 ( 3146.18 ) | 62.47 ( 47.46) | 5.97 ( 4.45 ) | <8.636×10^-5^ |
| Enuresis | Psychiatric disorders | 8 | 42.60 ( 21.27 - 85.33 ) | 42.48 ( 323.42 ) | 42.40 ( 21.17) | 5.41 ( 1.96 ) | <8.636×10^-5^ |
| Gastroenteritis viral | Infections and infestations | 34 | 41.60 ( 29.65 - 58.35 ) | 41.11 ( 1328.45 ) | 41.03 ( 29.25) | 5.36 ( 3.77 ) | <8.636×10^-5^ |
| Troponin increased | Investigations | 8 | 23.54 ( 11.75 - 47.13 ) | 23.47 ( 171.96 ) | 23.45 ( 11.71) | 4.55 ( 1.78 ) | <8.636×10^-5^ |
| Anger | Psychiatric disorders | 32 | 20.41 ( 14.40 - 28.93 ) | 20.20 ( 583.59 ) | 20.18 ( 14.24) | 4.33 ( 3.17 ) | <8.636×10^-5^ |
| Pharyngitis streptococcal | Infections and infestations | 10 | 20.05 ( 10.77 - 37.32 ) | 19.98 ( 180.20 ) | 19.97 ( 10.73) | 4.32 ( 2.00 ) | <8.636×10^-5^ |
| Emotional disorder | Psychiatric disorders | 28 | 19.65 ( 13.54 - 28.51 ) | 19.47 ( 490.26 ) | 19.45 ( 13.40) | 4.28 ( 3.03 ) | <8.636×10^-5^ |
| Weight increased | Investigations | 186 | 19.53 ( 16.83 - 22.66 ) | 18.33 ( 3055.10 ) | 18.31 ( 15.78) | 4.19 ( 3.85 ) | <8.636×10^-5^ |
| Mood altered | Psychiatric disorders | 19 | 15.25 ( 9.71 - 23.95 ) | 15.16 ( 251.14 ) | 15.15 ( 9.64) | 3.92 ( 2.50 ) | <8.636×10^-5^ |
| Abnormal behaviour | Psychiatric disorders | 27 | 14.62 ( 10.00 - 21.35 ) | 14.49 ( 338.99 ) | 14.48 ( 9.91) | 3.86 ( 2.74 ) | <8.636×10^-5^ |
| Ear infection | Infections and infestations | 14 | 11.64 ( 6.88 - 19.68 ) | 11.59 ( 135.42 ) | 11.58 ( 6.85) | 3.53 ( 2.02 ) | <8.636×10^-5^ |
| Aggression | Psychiatric disorders | 27 | 11.66 ( 7.98 - 17.04 ) | 11.56 ( 260.58 ) | 11.56 ( 7.91) | 3.53 ( 2.52 ) | <8.636×10^-5^ |
| Mood swings | Psychiatric disorders | 13 | 8.78 ( 5.09 - 15.15 ) | 8.75 ( 89.22 ) | 8.75 ( 5.07) | 3.13 ( 1.72 ) | <8.636×10^-5^ |
| Viral infection | Infections and infestations | 13 | 8.57 ( 4.97 - 14.78 ) | 8.54 ( 86.53 ) | 8.53 ( 4.95) | 3.09 ( 1.70 ) | <8.636×10^-5^ |
| Crying | General disorders and administration site conditions | 13 | 7.66 ( 4.44 - 13.21 ) | 7.63 ( 74.90 ) | 7.63 ( 4.42) | 2.93 ( 1.60 ) | <8.636×10^-5^ |
| Irritability | Psychiatric disorders | 21 | 7.55 ( 4.91 - 11.59 ) | 7.50 ( 118.36 ) | 7.50 ( 4.88) | 2.91 ( 1.91 ) | <8.636×10^-5^ |
| Pollakiuria | Renal and urinary disorders | 13 | 6.79 ( 3.94 - 11.71 ) | 6.76 ( 63.87 ) | 6.76 ( 3.92) | 2.76 ( 1.49 ) | <8.636×10^-5^ |
| Blood creatine phosphokinase increased | Investigations | 9 | 6.53 ( 3.39 - 12.56 ) | 6.51 ( 41.97 ) | 6.51 ( 3.38) | 2.70 ( 1.15 ) | <8.636×10^-5^ |
| Influenza | Infections and infestations | 31 | 6.19 ( 4.35 - 8.82 ) | 6.14 ( 133.48 ) | 6.14 ( 4.31) | 2.62 ( 1.89 ) | <8.636×10^-5^ |
| Gait inability | General disorders and administration site conditions | 9 | 6.15 ( 3.20 - 11.83 ) | 6.13 ( 38.69 ) | 6.13 ( 3.19) | 2.62 ( 1.10 ) | <8.636×10^-5^ |
| Muscular weakness | Musculoskeletal and connective tissue disorders | 31 | 5.98 ( 4.20 - 8.52 ) | 5.93 ( 127.12 ) | 5.92 ( 4.16) | 2.57 ( 1.85 ) | <8.636×10^-5^ |
| Swelling face | General disorders and administration site conditions | 17 | 5.70 ( 3.54 - 9.19 ) | 5.68 ( 65.55 ) | 5.68 ( 3.52) | 2.50 ( 1.49 ) | <8.636×10^-5^ |
| Mobility decreased | Musculoskeletal and connective tissue disorders | 16 | 4.98 ( 3.05 - 8.15 ) | 4.96 ( 50.67 ) | 4.96 ( 3.04) | 2.31 ( 1.31 ) | <8.636×10^-5^ |
| Abdominal discomfort | Gastrointestinal disorders | 37 | 4.96 ( 3.59 - 6.86 ) | 4.91 ( 115.49 ) | 4.91 ( 3.55) | 2.30 ( 1.68 ) | <8.636×10^-5^ |
| Nasal congestion | Respiratory, thoracic and mediastinal disorders | 12 | 4.52 ( 2.56 - 7.97 ) | 4.50 ( 32.74 ) | 4.50 ( 2.55) | 2.17 ( 1.02 ) | <8.636×10^-5^ |
| Abdominal pain upper | Gastrointestinal disorders | 36 | 3.87 ( 2.79 - 5.38 ) | 3.84 ( 75.78 ) | 3.84 ( 2.76) | 1.94 ( 1.36 ) | <8.636×10^-5^ |
| Nasopharyngitis | Infections and infestations | 32 | 3.83 ( 2.70 - 5.43 ) | 3.80 ( 66.20 ) | 3.80 ( 2.68) | 1.93 ( 1.30 ) | <8.636×10^-5^ |
| Weight decreased | Investigations | 44 | 3.46 ( 2.57 - 4.66 ) | 3.42 ( 75.68 ) | 3.42 ( 2.54) | 1.77 ( 1.26 ) | <8.636×10^-5^ |
| Asthenia | General disorders and administration site conditions | 58 | 3.39 ( 2.61 - 4.40 ) | 3.34 ( 95.69 ) | 3.34 ( 2.58) | 1.74 ( 1.30 ) | <8.636×10^-5^ |
| Vomiting | Gastrointestinal disorders | 66 | 3.12 ( 2.45 - 3.99 ) | 3.08 ( 93.11 ) | 3.07 ( 2.41) | 1.62 ( 1.22 ) | <8.636×10^-5^ |
| Condition aggravated | General disorders and administration site conditions | 40 | 2.96 ( 2.17 - 4.04 ) | 2.93 ( 51.13 ) | 2.93 ( 2.15) | 1.55 ( 1.03 ) | <8.636×10^-5^ |

**FAERS**: The U.S. FDA Adverse Event Reporting System; **Cases**: Number of reported cases; **PT**: Preferred Term; **ROR**: reporting odds ratio; **95%CI**: 95% Confidence Interval; **PRR**: proportional reporting ratio; **χ2**: chi-squared; **IC**: information component; **IC025**: Information Component 2.5th percentile; **EBGM**: empirical Bayes geometric mean; **EBGM05**: Empirical Bayes Geometric Mean 5th percentile; ***Bonferroni-P***: P-value after Bonferroni correction for multiple testing. The adjusted significance threshold was set at P < 8.636×10^-5^ based on 579 tested Preferred Terms.
